# To Remove Amalgams and Refill with Gold

**Published:** 1863-07

**Authors:** Geo. F. Foote


### TO REMOVE AMALGAMS AND REFILL WITH GOLD.

BY GEO. F. FOOTE.

This is no ordinary operation, and so long as this bane to some of the profession is an adjunct in the practice with so many others of its members, it is well for those who think they are educated to a stand point beyond its use, to know how they can best bring their patients into a physical condition corresponding to their own ideas of utility and beauty.

The removing of an amalgam is oftentimes a long and tedious process, on account of the size and density of the filling and fragility of the tooth. The drill, gouge and chisel are the principle instruments, and in many cases we have been able to soften by the use of crude mercury. To excite a prompt action of the mercury some have recommended the use of sulphuric acid to first brighten the surface of the filling, but with a sharp instrument the surface is readily made bright and clean without endangering the tooth bone to the disorganizing effect of an acid. Great care in the manipulation is necessary to avoid fracturing the tooth.

Every vestage of the amalgum should be removed and the disintegrated tooth bone beneath it carefully cleaned away and the cavity and fangs thoroughly washed with a syringe, using tepid water. I include the fangs, for in most cases the nerve is dead, and the nerve cavities require filling.

This washing should be repeated daily for several days before refilling, by which means the stimulating effects of the water brings on a healthy reaction, and the danger of periostial inflammation is almost entirely removed; while at the same time the tooth is more or less bleached, and the offensive color, produced by the amalgam, nearly removed. It is not a safe practice to refill immediately with gold without this treatment, as there is danger of inflammation and abscess.

The danger arises from substituting a tight filling for one that was sufficiently porous in its texture, or from the deoxydised surface in contact with the tooth bone, gave sufficient space for the escape of the gasses arising from the decomposition of the soft parts within, as well as by endosmose and exosmose, a free transmission of fluids. I have often observed that but few alveolar abscesses with an opening through the gums are found in connection with amalgam fillings. This must arise from the imperfection of the material used in filling in those cases certainly where purulent matter is found in the pulp cavity, which is quite common in all large amalgams.

Gasses arising from the decomposition of the soft parts within must find an exit, and if it does not escape through an improperly filled cavity, nature kindly opens a passage for it through the alveolar process and gum, sometimes it follows the fang, escaping by the side of it.

Therefore, it is highly important to make sure of a healthy condition of the internal portion of the tooth before substituting a perfect filling. The application of water is the only additional treatment needed. Wash thoroughly, wash daily, and nature will rally as the poison of the virus is removed, to restore the parts to a normal condition, when a gold filling may be introduced with almost a certainty of success.

The necessity for removing these amalgams is of course a mooted point and will continue so long as there remains a want for something cheaper than gold for filling teeth. That something to take the place of gold in many cases of common practice is wanted, is apparent to most practitioners. But until something better is discovered it seems to me that we had better confine ourselves to gutta percha, tin foil or Wood's stopping rather than to so doubtful a material as an amalgam.

Of these I have removed a great many—and in nearly every case I have found the parts beneath in a state calling loudly for a change. It is often the case that those whose surfaces were the most fair presented a foul and disintegrated state within.

Of course, in my own mind, there is but one opinion based on my own experience in addition to other lights, and I must, as I have heretofore done, continue the removal of these, to me, offensive sights, whenever they are presented.

This I do with all due respect to my professional brethren. It is a matter where we may differ in opinion and differ conscientiously. Each one of us must be left at liberty to pursue that course which may seem to him best calculated to promote the welfare of his patients, and to elevate him and his profession.

